# Prevalence, Virulence Genes and Antimicrobial Resistance Profiles of *Salmonella* Serovars from Retail Beef in Selangor, Malaysia

**DOI:** 10.3389/fmicb.2017.02697

**Published:** 2018-01-11

**Authors:** Tze Y. Thung, Son Radu, Nor A. Mahyudin, Yaya Rukayadi, Zunita Zakaria, Nurzafirah Mazlan, Boon H. Tan, Epeng Lee, Soo L. Yeoh, Yih Z. Chin, Chia W. Tan, Chee H. Kuan, Dayang F. Basri, Che W. J. Wan Mohamed Radzi

**Affiliations:** ^1^Department of Food Science, Faculty of Food Science and Technology, Universiti Putra Malaysia, Serdang, Malaysia; ^2^Laboratory of Food Safety and Food Integrity, Institute of Tropical Agriculture and Food Security (ITAFoS), Universiti Putra Malaysia, Serdang, Malaysia; ^3^Department of Veterinary Pathology and Microbiology, Faculty of Veterinary Medicine, Universiti Putra Malaysia, Serdang, Malaysia; ^4^Department of Diagnostic and Allied Science, Faculty of Health and Life Science, Management and Science University, Shah Alam, Malaysia; ^5^Division of Applied Biomedical Sciences and Biotechnology, School of Health Sciences, International Medical University, Kuala Lumpur, Malaysia; ^6^Department of Agricultural and Food Science, Faculty of Science, Universiti Tunku Abdul Rahman, Kampar, Malaysia; ^7^Novel Antibiotic Laboratory, School of Diagnostic and Applied Health Sciences, Faculty of Health Sciences, Universiti Kebangsaan Malaysia, Kuala Lumpur, Malaysia; ^8^Department of Science and Technology Studies, Faculty of Science, University of Malaya, Kuala Lumpur, Malaysia

**Keywords:** beef meat, *Salmonella*, multiplex PCR, prevalence, antimicrobial resistance, virulence gene

## Abstract

The aim of the present study was to investigate the prevalence of *Salmonella* spp., *Salmonella* Enteritidis and *Salmonella* Typhimurium in retail beef from different retail markets of Selangor area, as well as, to assess their pathogenic potential and antimicrobial resistance. A total of 240 retail beef meat samples (chuck = 60; rib = 60; round = 60; sirloin = 60) were randomly collected. The multiplex polymerase chain reaction (mPCR) in combination with the most probable number (MPN) method was employed to detect *Salmonella* spp., *S*. Enteritidis and *S*. Typhimurium in the meat samples. The prevalence of *Salmonella* spp., *S*. Enteritidis and *S*. Typhimurium in 240 beef meat samples were 7.50, 1.25, and 0.83%, respectively. The microbial loads of total *Salmonella* was found in the range of <3 to 15 MPN/g. Eight different serovars of *Salmonella* were identified among the 23 isolates, and *S*. Agona was the predominant serovar (26.09%). Interestingly, all the *Salmonella* isolates were resistant to penicillin, erythromycin and vancomycin, but the sensitivity was observed for tetracycline, gentamicin and amoxicillin/clavulanic acid. All 23 isolates were resistant to at least three antibiotics. Two *S*. Typhimurium isolates (8.70%) exhibited the highest multiple antibiotic resistance (MAR) index value of 0.56 which shown resistance to nine antibiotics. PCR analysis of virulence genes showed that all *Salmonella* isolates (100%) were positive for the *invA* gene. Meanwhile, *pefA* was only identified in *S*. Enteritidis and *S*. Typhimurium. The findings in this study indicate that retail beef products tested were widely contaminated with multi-drug resistant (MDR) *Salmonella* and various virulence genes are present among the isolated *Salmonella* serovars.

## Introduction

The intestinal epithelium infection known as salmonellosis is caused by the genus *Salmonella*. The major pathogenic serovars of *Salmonella enterica* that infect humans from a variety of different food products include *Salmonella* Enteritidis and *Salmonella* Typhimurium (Kramarenko et al., 2014; Yang et al., 2016; Ed-dra et al., 2017). In the USA over 40,000 salmonellosis cases are reported each year and foods of animal origin are considered to be the most likely source of *Salmonella* (Finstad et al., 2012). It is well known that human *Salmonella* infections are associated with many different kinds of food, including beef meat and beef meat products (Brichta-Harhay et al., 2008; Sallam et al., 2014). Hence, the presence of *Salmonella* in beef at the slaughter level and at the market is a significant food safety risk.

Sensitive and specific methods with shorter turnaround time for the detection and identification of *Salmonella* are needed to reduce testing-related laboratory costs. Therefore, multiplex polymerase chain reaction (mPCR) uses few pairs of primers simultaneously detecting different pathogens in the same samples has the potential as a reliable and effective method (Pui et al., 2011). Previously, a detection method consist of mPCR and the most probable number (MPN) method has successfully been performed to detect and identify *S*. Enteritidis and *S*. Typhimurium in chicken meat (Thung et al., 2016). Indeed, the mPCR-MPN method has widely been used to detect and enumerate food-borne pathogens such as *Campylobacter* spp. (Chai et al., 2007), *Listeria monocytogenes* (Kuan et al., 2013) and *Vibrio parahaemolyticus* (Tan et al., 2017). Recently, gold nanoparticle-aptamer-based localized surface plasmon resonance (LSPR) sensing chip was developed to enable the ultra-sensitive and selective detection of *S*. Typhimurium in pork meat (Oh et al., 2017).

In general, bacterial virulence factors have a crucial role for systemic infections. The pathogenicity of *Salmonella* strains has been related to numerous virulence genes present in the chromosomal *Salmonella* pathogenicity islands (SPIs) (Nayak et al., 2004). Genes such as *invA* and *hilA*, found in SPI, allow *Salmonella* to invade epithelial cells (Cardona-Castro et al., 2002; Nayak et al., 2004). Besides, *Salmonella* outer proteins (sops) (SPI effector protein) encoded by *sop* gene have relevance to *Salmonella* virulence (Huehn et al., 2010). Meanwhile, the plasmid encoded fimbriae (*pefA*) gene contributes to the adhesion of *Salmonella* to epithelial cells (Murugkar et al., 2003). Other chromosomal gene like *stn*, codes for enterotoxin production has been shown to be a causative agent of diarrhea (Huehn et al., 2010). In addition, virulence plasmids carrying virulence genes such as the *spv* operon (*Salmonella* plasmid virulence) contribute to the colonization of deeper tissues among other functions (Swamy et al., 1996).

To date, the emergence and spread of antimicrobial resistance among zoonotic *Salmonella* has become a public health threat (Sallam et al., 2014). Importantly, *Salmonella* strains having “clinically important resistance” to some agents like extended-spectrum cephalosporins and fluoroquinolones, have been isolated from livestock (Li et al., 2013). In most developing countries, misuse and overuse of antibiotics has contributed to the increasing trend of multi-resistance in *Salmonella* (Ed-dra et al., 2017). In Selangor (center of Peninsular Malaysia), although some reports were found based on the prevalence of *Salmonella* in different types of foods, but limited information on the surveillance study of *Salmonella* spp., *S*. Enteritidis and *S*. Typhimurium in beef meat at retail level are available. Therefore, the aim of this study was to assess the prevalence, virulence genes and antimicrobial resistance of *Salmonella* serovars isolated from retail beef meat in Selangor area.

## Materials and methods

### Collection of meat sample

Four different parts of beef meat samples (chuck = 60; rib = 60; round = 60; sirloin = 60) with a total number of 240 were collected from retail markets (wet markets and hypermarkets) in Selangor area over 9 months period from September 2014 to May 2015 (approximately 25 to 27 samples per month). The retail meat samples were kept in sterile stomacher bags and transferred to the laboratory for further analysis.

### Enrichment and most probable number (MPN) method

Ten gram of meat sample was homogenized for 50 s using a stomacher after added in 90 mL of sterile buffered peptone water (BPW) (Merck, Darmstadt, Germany). Then, the suspension was diluted up to 1,000-fold with 10-fold serial. Later, MPN method (three-tube) was performed by transferring 1 mL of each dilution into three replicate tubes with 10 mL of Rappaport-Vasiliadis (RV) broth (Merck, Darmstadt, Germany) each, and followed by overnight incubation at 37°C under aerobic conditions. The turbid MPN tubes were selected for subsequent DNA extraction using the boiled-cell method as described previously (Chai et al., 2007).

### Multiplex PCR conditions

For mPCR detection, three primer pairs were used to identify randomly selected-sequence of unknown function gene (429 bp) for *Salmonella* spp., *sdfI* gene (304 bp) for *S*. Enteritidis, and *fliC* gene (620 bp) for *S*. Typhimurium. The sequences of the primer pair used for targeting random sequence were 5′-GCCAACCATTGCTAAATTGGCGCA-3′ and 5′-GGTAGAAATTCCCAGCGGGTACTGG-3′ (Soumet et al., 1999), whereas the primer pair used for targeting the *sdfI* gene were 5′-TGTGTTTTATCTGATGCAAGAGG-3′ and 5′-TGAACTACGTTCGTTCTTCTGG-3′ (Alvarez et al., 2004), followed by the primer pair used for targeting the *fliC* gene were 5′-CGGTGTTGCCCAGGTTGGTAAT-3′ and 5′-ACTGGTAAAGATGGCT-3′ (Soumet et al., 1999). We optimized the mPCR reaction conditions by a series of preliminary experiments so that the three independent PCR reactions can be performed in the same tube with the detection limit of 10^5^ cfu/mL (data not shown). The optimized mPCR reaction mixture (25 μL) contained 2 μL of DNA template, 5 μL of 5 × PCR buffer, 2.5 μL of 25 mM MgCl_2_, 0.5 μL of 10 mM deoxynucleotide triphosphate (dNTP), 0.5 μL of 1.2 μM primer mix and 14.2 μL of deionized water. The mixture was then treated with 0.3 μL (1.5 U) *Taq* DNA polymerase. PCR amplification was performed in triplicate with the following conditions: initial denaturation at 94°C for 2 min, 30 cycles of denaturation at 94°C for 45 s, annealing at 53°C for 1 min, extension at 72°C for 1 min and final extension at 72°C for 7 min. The positive controls used were *S*. Typhimurium ATCC 14028 and *S*. Enteritidis ATCC 13076. *Escherichia coli* ATCC 25922 was used as a negative control.

### Isolation and identification of *Salmonella*

The turbid MPN tubes were confirmed to be *Salmonella* by plating on selective CHROMagar *Salmonella* (CHROMagar Microbiology, Paris, France) and Xylose Lysine Deoxycholate (XLD) (Merck, Darmstadt, Germany) agar plates, and incubated at 37°C for 24 h. All the *Salmonella* isolates were then serotyped by slide agglutination using polyvalent “O” and “H” antisera (BD, Franklin Lakes, USA) at Veterinary Research Institute (VRI), Ipoh, Malaysia in accordance with the Kauffmann-White scheme.

### Antimicrobial resistance profiles

The antimicrobial susceptibility was evaluated according to Clinical and Laboratory Standarts Institude (2012) by using disc diffusion method. Briefly, isolates were cultured aerobically in 10 mL Mueller-Hinton (MH) broth (Merck, Darmstadt, Germany) at 37°C for 24 h. Overnight cultures, grown on MH broth (OD adjusted to 0.5 MacFarland unit), were swabbed evenly with sterile non-toxic cotton swab on MH agar plates and left to dry for 2 to 4 min. Then, antimicrobial sensitivity discs were placed on the culture by using a disk dispenser and incubated at 37°C for 24 h. The tested antimicrobials were amoxicillin/clavulanic acid (AMC, 30 μg), amoxycillin (AML, 30 μg), ceftazidime (CAZ, 30 μg), cephazolin (KZ, 30 μg), ciprofloxacin (CIP, 5 μg), erythromycin (E, 15 μg), chloramphenicol (C, 30 μg), ampicillin (AMP, 10 μg), penicillin (P, 10 μg), streptomycin (S, 10 μg), tetracycline (TE, 30 μg), kanamycin (K, 30 μg), gentamicin (CN, 10 μg), vancomycin (VA, 30 μg), nalidixic acid (NA, 30 μg), and suphamethoxazole/trimethoprim (SXT, 25 μg) (Oxoid, Hamphire, United Kingdom). The multiple antibiotic resistance (MAR) index was calculated as “a/b,” where “a” the number of antibiotics for a particular isolate was resistant and “b” the total number of antibiotics tested (Krumperman, 1983).

### Detection of virulence genes

All *Salmonella* isolates collected in this study were screened for the presence of virulence genes using PCR. The primers, the size in base pairs of the respective amplification products and the references used for detection of six virulence genes are presented in Table 1. The virulence genes under study were *invA, pefA, hilA, sopB, stn*, and *spvC*. Positive (*S*. Typhimurium ATCC 14028 and *S*. Enteritidis ATCC 13076) and negative control (*E. coli* ATCC 25922) were conducted in the detection procedure. To evaluate the reproducibility of the experiments, PCR amplification and electrophoresis experiments were carried out in triplicate.

**Table 1:** PCR primers used for amplification of virulence genes in *Salmonella* isolates.

**Virulence genes**	**Primer sequence (5′-3′)**	**Size (bp)**	**Reference**
*invA*	F- TATCGCCACGTTCGGCAA	275	Nayak et al., 2004
*pefA*	R- TCGCACCGTCAAAGGAACC	700	Murugkar et al., 2003
*hilA*	F- TGTTTCCGGGCTTGTGCT	854	Cardona-Castro et al., 2002
*sopB*	R- CAGGGCATTTGCTGATTCTTCC	517	Huehn et al., 2010
*stn*	F- CGGAAGCTTATTTGCGCCATGCTGAGGTAG	617	Murugkar et al., 2003
*spvC*	R- GCATGGATCCCCGCCGGCGAGAT TGTG	669	Swamy et al., 1996
	F- TCAGAAGRCGTCTAACCACTC		
	R- TACCGTCCTCATGCACACTC		
	F- TTGTGTCGCTATCACTGGCAACC		
	R- ATTCGTAACCCGCTCTCGTCC		
	F- CGGAAATACCATCAAATA		
	R- CCCAAACCCATACTTACTCTG		

### Statistical analysis

All measurements were carried out in triplicate. Minitab (v. 14) statistical package (Minitab Inc., State College, PA) was used to determine if there was any significant difference between the prevalence of *Salmonella* in beef meat from wet market and hypermarket. For all analysis, *P* < 0.05 was considered significant.

## Results

### Prevalence of *Salmonella* in beef

**Figure 1:**
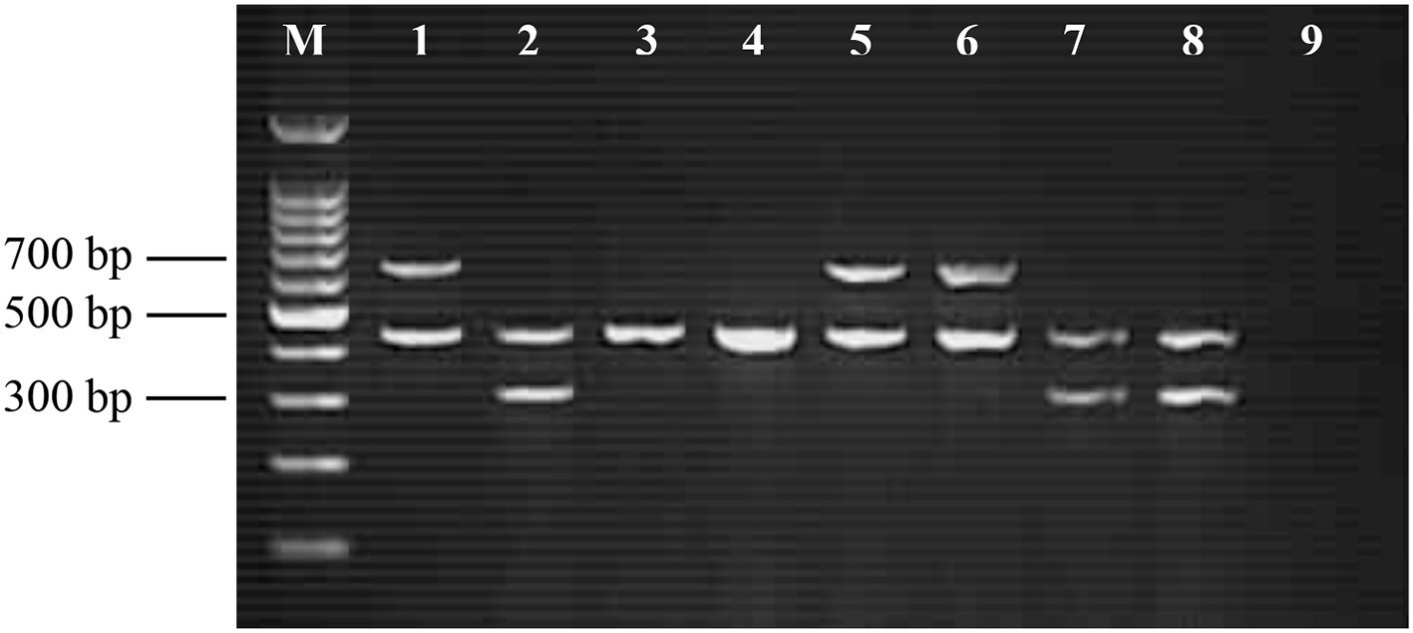
Representative amplification of random sequence, *sdfI* gene and *fliC* gene for identification of *Salmonella* spp. (429 bp), *S*. Enteritidis (304 bp) and *S*. Typhimurium (620 bp), respectively. Lane M: 100-bp DNA ladder; Lane 1: mixture of *Salmonella* spp. and *S*. Typhimurium (positive); Lane 2: *Salmonella* spp. and *S*. Enteritidis (positive); Lane 3 to Lane 8: meat samples examined; and Lane 9: negative control.

The target genes specific to *Salmonella* spp., *S*. Enteritidis and *S*. Typhimurium produced amplicons at 429, 304, and 620 bp, respectively. Figure 1 shows the result of gel electrophoresis comparing various combinations of the PCR primer sets and verifying the mPCR established for the current study consists of three independent and specific PCR reactions. Of the 240 retail beef meat samples tested, the contamination rates were 7.50% (*n* = 18), 1.25% (*n* = 3), and 0.83% (*n* = 2) for *Salmonella* spp., *S*. Enteritidis and *S*. Typhimurium, respectively (Table 2). Beef part round was the major reservoir for *Salmonella*, with prevalence rate of 16.67% (*n* = 60). The prevalence of *Salmonella* spp. in wet markets (10.00%) were significantly higher than hypermarkets (5.00%) (*P* < 0.05). As shown in Table 3, the highest microbial loads of *Salmonella* was found in *Salmonella* spp. (15.0 MPN/g), followed by *S*. Enteritidis (3.6 MPN/g) and *S*. Typhimurium (3.6 MPN/g).

**Table 2:** Prevalence of *Salmonella* spp., *Salmonella* Enteritidis and *Salmonella* Typhimurium in beef meat samples using MPN-mPCR method.

**Beef part**	**Wet markets**				**Hypermarkets**			
	**No. of positive sample**				**No. of positive sample**			
	***n*^a^**	***Salmonella* spp**.	***S*. Enteritidis**	***S*. Typhimurium**	***n***	***Salmonella* spp**.	***S*. Enteritidis**	***S*. Typhimurium**
Chuck	30	3 (10.0%)^b^	0 (0.0%)	0 (0.0%)	30	2 (6.7%)	0 (0.0%)	0 (0.0%)
Rib	30	3 (10.0%)	0 (0.0%)	0 (0.0%)	30	0 (0.0%)	0 (0.0%)	0 (0.0%)
Round	30	4 (13.3%)	1 (3.3%)	1 (3.3%)	30	2 (6.7%)	1 (3.3%)	1 (3.3%)
Sirloin	30	2 (6.7%)	1 (3.3%)	0 (0.0%)	30	2 (6.7%)	0 (0.0%)	0 (0.0%)
Total	120	12 (10.0%)	2 (1.7%)	1 (0.8%)	120	6 (5.0%)	1 (0.8%)	1 (0.8%)

a *Number of samples*.

b *Percentage of positive samples*.

**Table 3:** Microbial loads of *Salmonella* spp., *Salmonella* Enteritidis and *Salmonella* Typhimurium (MPN/g) in beef meat samples using MPN-mPCR method.

**Beef part**	**Wet markets**									**Hypermarkets**								
	***Salmonella*** **spp**.			***S***. **Enteritidis**			***S***. **Typhimurium**			***Salmonella*** **spp**.			***S***. **Enteritidis**			***S***. **Typhimurium**		
	**Min^a^**	**Med^b^**	**Max^c^**	**Min**	**Med**	**Max**	**Min**	**Med**	**Max**	**Min**	**Med**	**Max**	**Min**	**Med**	**Max**	**Min**	**Med**	**Max**
Chuck	<3	<3	7.4	<3	<3	<3	<3	<3	<3	<3	<3	3.6	<3	<3	<3	<3	<3	<3
Rib	<3	<3	7.4	<3	<3	<3	<3	<3	<3	<3	<3	<3	<3	<3	<3	<3	<3	<3
Round	<3	<3	15	<3	<3	3.6	<3	<3	3.6	<3	<3	3.6	<3	<3	3.6	<3	<3	3.6
Sirloin	<3	<3	3.6	<3	<3	3.6	<3	<3	<3	<3	<3	3.6	<3	<3	<3	<3	<3	<3

a *Minimum MPN/g value*.

b *Median MPN/g value*.

c *Maximum MPN/g value*.

### Antimicrobial resistance profiles

Antibiotic sensitivity testing was performed for the 23 isolated *Salmonella* strains, which included *S*. Enteritidis (*n* = 3), *S*. Typhimurium (*n* = 2), *S*. Agona (*n* = 6), *S*. Anatum (*n* = 3), *S*. London (*n* = 3), *S*. Newport (*n* = 3), *S*. Stanley (*n* = 1), and *S*. Weltevreden (*n* = 2). As shown in Table 4, three antibiotics gentamicin, amoxicillin/clavulanic acid, and tetracycline were effective (100%) to all isolates. In this study, resistance to erythromycin, penicillin and vancomycin were seen in 100% of *S*. Enteritidis, *S*. Typhimurium, *S*. Agona, *S*. Anatum, *S*. London, *S*. Newport, *S*. Stanley and *S*. Weltevreden isolates. As shown in Table 5, the highest MAR index value of 0.56 was found in two *S*. Typhimurium isolates. Observation from the presence study indicated that all *Salmonella* isolates were multi-drug resistant (MDR) strains, which showed resistance to three antibiotics (penicillin, vancomycin and erythromycin) or more. Nine *Salmonella* isolates (39.13%) mainly from *S*. Agona and *S*. Newport were resistant to four antibiotics, and three *S*. Enteritidis (13.04%) were resistant to at least five of the antibiotics.

**Table 4:** Antimicrobial susceptibility pattern of *Salmonella* isolates.

**Antimicrobial agent**	**No. of isolates tested**	**Antibiogram pattern of** ***Salmonella*** **isolates**		
		**Resistant (%)**	**Intermediate (%)**	**Sensitive (%)**
Amoxycillin (30 μg)	23	4 (17.39)	4 (17.39)	15 (65.22)
Ampicillin (10 μg)	23	11 (47.83)	8 (34.78)	4 (17.39)
Amoxicillin/Clavulanic acid (30 μg)	23	–	–	23 (100)
Cephazolin (30 μg)	23	2 (8.70)	2 (8.70)	19 (82.60)
Ceftazidime (30 μg)	23	–	5 (21.74)	18 (78.26)
Chloramphenicol (30 μg)	23	7 (30.43)	13 (56.52)	3 (13.05)
Ciprofloxacin (5 μg)	23	5 (21.74)	5 (21.74)	13 (56.52)
Erythromycin (15 μg)	23	23 (100)	–	–
Gentamicin (10 μg)	23	–	–	23 (100)
Kanamycin (30 μg)	23	–	2 (8.70)	21 (91.30)
Penicillin (10 μg)	23	23 (100)	–	–
Nalidixic acid (30 μg)	23	–	2 (8.70)	21 (91.30)
Streptomycin (10 μg)	23	–	2 (8.70)	21 (91.30)
Suphamethoxazole/trimethoprim (25 μg)	23	4 (17.39)	6 (26.09)	13 (56.52)
Tetracycline (30 μg)	23	–	–	23 (100)
Vancomycin (30 μg)	23	23 (100)	–	–

**Table 5:** The antibiotic resistance profile patterns and multiple antibiotic resistance (MAR) index of *Salmonella* isolates.

**Isolate no**.	**Retail market**	**Source**	***Salmonella* serovar**	**Antibiotic resistance profiles^a^**	**MAR index**
1	Wet market	Round	*S*. Enteritidis	AMP, C, E, P, SXT, VA	0.38
2	Wet market	Sirloin	*S*. Enteritidis	AMP, C, E, P, VA	0.31
3	Wet market	Round	*S*. Typhimurium	AML, AMP, KZ, C,CIP, E, P, SXT, VA	0.56
4	Wet market	Chuck	*S*. Agona	AMP, E, P, VA	0.25
5	Wet market	Rib	*S*. Agona	AMP, E, P, VA	0.25
6	Wet market	Rib	*S*. Agona	AMP, E, P, VA	0.25
7	Wet market	Rib	*S*. Agona	AMP, E, P, VA	0.25
8	Wet market	Round	*S*. Anatum	E, P, VA	0.19
9	Wet market	Round	*S*. Anatum	E, P, VA	0.19
10	Wet market	Sirloin	*S*. Anatum	E, P, VA	0.19
11	Wet market	Chuck	*S*. London	E, P, VA	0.19
12	Wet market	Chuck	*S*. London	E, P, VA	0.19
13	Wet market	Round	*S*. Newport	CIP, E, P, VA	0.25
14	Wet market	Round	*S*. Newport	CIP, E, P, VA	0.25
15	Wet market	Sirloin	*S*. Stanley	E, P, VA	0.19
16	Hypermarket	Round	*S*. Enteritidis	AMP, C, E, P, SXT, VA	0.38
17	Hypermarket	Round	*S*. Typhimurium	AML, AMP, KZ, C, CIP, E, P, SXT, VA	0.56
18	Hypermarket	Chuck	*S*. Agona	AMP, E, P, VA	0.25
19	Hypermarket	Round	*S*. Agona	AMP, E, P, VA	0.25
20	Hypermarket	Chuck	*S*. London	E, P, VA	0.19
21	Hypermarket	Round	*S*. Newport	CIP, E, P, VA	0.25
22	Hypermarket	Sirloin	*S*. Weltevreden	AML, C, E, P, VA	0.31
23	Hypermarket	Sirloin	*S*. Weltevreden	AML, C, E, P, VA	0.31

a *AML, Amoxycillin; AMP, Ampicillin; KZ, Cephazolin; C, Chloramphenicol; CIP, Ciprofloxacin; E, Erythromycin; P, Penicillin; SXT, Suphamethoxazole/trimethoprim; VA, Vancomycin*.

### Distribution of virulence genes among *Salmonella* isolates

All 23 *Salmonella* isolates were tested by PCR for the presence of virulence genes. The invasion gene operon *invA* was detected in all *Salmonella* isolates (Table 6). Regarding the different frequencies of *hilA, sopB*, and *stn* genes among different serovars, a clear difference was noticed in the occurrence of these genes among the isolates; *S*. London and *S*. Stanley did not show the presence of *sopB* gene. Furthermore, *pefA* gene was present in 3 of the 23 isolates tested (13.04%), comprising *S*. Enteritidis (one isolate) and *S*. Typhimurium (two isolates). Overall, the serovars tested showed at least three virulence-associated genes.

**Table 6:** Virulence genes profiles of *Salmonella* isolates.

**Gene**	**Number of** ***Salmonella*** **serovars positive for virulence genes**								**Total (%) (23)**
	***S*. Enteritidis (3)**	***S*. Typhimurium (2)**	***S*. Agona (6)**	***S*. Anatum (3)**	***S*. London (3)**	***S*. Newport (3)**	***S*. Stanley (1)**	***S*. Weltevreden (2)**	
*invA*	3	2	6	3	3	3	1	2	23 (100)
*pefA*	1	2	0	0	0	0	0	0	3 (13.04)
*hilA*	3	2	4	3	3	1	1	2	19 (82.61)
*sopB*	2	1	6	1	0	2	0	2	14 (60.87)
*stn*	3	1	3	1	2	3	1	2	16 (69.57)
*spvC*	0	0	0	0	0	0	0	0	0 (0)

## Discussion

Results of investigations of retail beef meat samples do provide an estimate of the prevalence of *Salmonella* in retail shops. The high incidence of *Salmonella* in wet markets of the present study indicated poor sanitary condition in the food processing environment and lack of better personal hygiene of food handlers during product preparation. High incidence of *Salmonella* in wet markets was observed in the previous study of Thung et al. (2016), who found higher prevalence of *Salmonella* in retail chicken meat samples. An additional factor underlying potential differences in prevalence between wet markets and hypermarkets was that the storage temperature of the samples (Donado-Godoy et al., 2012). Meanwhile, the presence of *Salmonella* in retail beef meat might be due to the production system and conditions, hygienic slaughter, and transport before sale. In this study, the incidence of *Salmonella* in beef meat samples (9.58%, *n* = 240) was higher than the incidence (2.16%, *n* = 417) in Poland (Wieczorek and Osek, 2013) and less than the incidence (39.87%, *n* = 158) in North Vietnam (Thai et al., 2012). This could be due to the geographical variation such as climate and feed. On the other hand, prevalence of *Salmonella* has been reported in other food products in Malaysia. For example, *Salmonella* spp. and *S*. Typhimurium were detected in sliced fruits (such as papaya, watermelon, mango, sapodilla, jackfruit, dragon fruit and honeydew) (Pui et al., 2011), and vegetables (such as cabbage, carrot, capsicum, cucumber, lettuce and tomato) (Elexson et al., 2011). Besides, Najwa et al. (2015) have shown that *Salmonella* spp., *S*. Typhimurium and *S*. Enteritidis were detected in different types of local salad known as *ulam* (such as *kacang botol, kacang panjang, pegaga nyonya*, and *selom*).

In the present study, a combined MPN-mPCR was used to quantify the microbial load (MPN/g) which can facilitate the enumeration of *Salmonella* spp., *S*. Enteritidis and *S*. Typhimurium in the meat samples within a short period. The molecular amplification techniques can overcome the limitation of detecting viable but non-culturable (VBNC) cells with providing high specificity and sensitivity (Pui et al., 2011). The procedure of the MPN-mPCR should be familiar to laboratory personnel since it has been extensively used in academic research as well as in industrial settings. Previous studies have described detection methods that successfully combined MPN with mPCR to enumerate bacteria such as *Campylobacter, Listeria monocytogenus*, and *Vibrio parahaemolyticus* in samples (Kuan et al., 2017; Premarathne et al., 2017; Tan et al., 2017). Worth to note that beef meat products by their nature, undergo extensive processing and handling during their production, may also increase the risk of contamination (Thung et al., 2016). Typically, improper or ineffective cleaning of chopping boards, tables and knives does play a role in harboring and multiplying the organism. Cross-contamination may occur when microorganisms are transferred from one surface to another, possibly leading to contamination of other safe meat or clean equipment. *Salmonella* contamination was common in retail meats such as beef, pork and lamb, which could be a potential vehicle for transmitting *Salmonella* to humans (Yang et al., 2010). Thus, implementation and maintenance of some control measures like the good manufacturing practices (GMP) and hazard analysis and critical control point (HACCP), as well as further strengthening the education of food processors will be necessary, for reducing *Salmonella* contamination.

Due to clinical significance, determining or *Salmonella* resistance or otherwise to antimicrobial agents is critical for treatment during outbreaks. High resistance of *Salmonella* isolates to erythromycin, penicillin and vancomycin in this finding are of clinical concern and could be the result of widespread use of these antibiotics in Selangor area. Similarly, high percentage of penicillin and erythromycin resistance were observed in different *Salmonella* serovars which isolated from retail meat products such as beef burger, ground beef and fresh beef (Sallam et al., 2014). Interestingly, there were no *Salmonella* serovars resistant to amoxicillin/clavulanic acid, ceftazidime, gentamicin, kanamycin, nalidixic acid, streptomycin, and tetracycline. In contrast, resistance to tetracycline was observed among the serovars of *S*. Enteritidis and *S*. Typhimurium isolated from retail beef meat samples (Yang et al., 2010). Previously, a study in China demonstrated that all the *Salmonella* strains (*n* = 83) were sensitive to amoxicillin/clavulanic acid, while 98.80 and 92.77% were observed for gentamicin and tetracycline, respectively (Dong et al., 2014). In fact, MDR *Salmonella* serovars are considered to be more virulent than non-MDR *Salmonella* (Nayak et al., 2004; Dong et al., 2014). High percentages of antimicrobial resistant *Salmonella* serovars from retail meat products have been reported worldwide by several researchers (Yang et al., 2010; Thai et al., 2012; Sallam et al., 2014). In Malaysia, such observation was also reported by Geidam et al. (2012), where they were able to detect MDR *Salmonella* in the poultry environment in Selangor region. In this study, MDR *Salmonella* isolates are prevalent in both retail markets. Hence, more attention should be focused on the supervision and control of antimicrobial use, typically in the agriculture and human health care sectors in Malaysia.

Accordingly, the virulence of bacteria is influenced by both antimicrobial resistance and the presence of virulence genes (Huehn et al., 2010; Dong et al., 2014). The emergence of MDR strains of *Salmonella* are mainly based on the factors of genetic and biochemical mechanisms in order to enhance their survivability by preserving their drug resistance genes (Yang et al., 2010). Regarding the virulence factors that were analyzed, *S*. Enteritidis, *S*. Typhimurium, *S*. Agona, *S*. Anatum, *S*. Newport, and *S*. Weltevreden isolates showed a broader range of pathogenicity determinants as compared to other serovars. The most common virulence gene which present in *Salmonella, invA* gene, was used as PCR target gene for detection of *Salmonella* (Nayak et al., 2004; Dong et al., 2014). On the other hand, an OmpR/ToxR transcriptional regulator encoded by the *hilA* gene to activate the expression of invasion genes was shown to play an important role in *Salmonella* virulence (Cardona-Castro et al., 2002). In this study, the PCR screening using *hilA*-targeted *Salmonella*-specific primers showed a clear abundance of this virulence gene was detected in 19 of 23 analyzed strains (82.61%), irrespective of their serovars. In addition, similar results have been reported by Murugkar et al. (2003) who found that the chromosomally encoded virulent *stn* gene was widely distributed in all the isolated serovars. This strengthens to our present finding; the *stn* gene was prevalent among the isolated *Salmonella* serovars by PCR-based assay (69.57%). Several studies have shown that the *Salmonella* virulence plasmid plays an important role in human disease (Swamy et al., 1996).

In summary, this study has shown that a combined MPN-mPCR method was a reliable and useful for rapid screening of *Salmonella* from retail beef meat. Our results indicate that retail beef meat act as reservoirs in harboring multiple *Salmonella* serovars, where cross-contamination might be occurred during processing and at the retail level. *S*. Agona was the most common serotype found in retail beef. Moreover, the recovered *Salmonella* isolates exhibiting multi-drug resistant and multiple virulence genes, which constitute a possible risk to humans from consumption of these products. Therefore, it is important to manage the use of antimicrobial agents in livestock now, to prevent the acquisition and increased resistance to recent molecules in order to fight against the vertical and horizontal transfer of MDR strains. Alternatively, it is necessary for developing more effective intervention strategies such as green control method using bacteriophages as controlling measure in the food chain in order to reduce the risk of food-borne diseases.

## Author contributions

All authors listed have made a substantial, direct and intellectual contribution to the work, and approved it for publication.

### Conflict of interest statement

The authors declare that the research was conducted in the absence of any commercial or financial relationships that could be construed as a potential conflict of interest. The reviewer GP and handling Editor declared their shared affiliation.
